# Virtual interview training for neurosurgery applicants

**DOI:** 10.1016/j.amsu.2022.104013

**Published:** 2022-06-17

**Authors:** Susruta Manivannan, Sabbur K. Anwar, Ali Bakhsh, Conor S. Gillespie, Nick Carleton-Bland

**Affiliations:** a Department of Neurosurgery, Southampton General Hospital, Southampton, UK; b Department of Surgery, Northampton General Hospital, Northampton, UK; c Department of Neurosurgery, The Walton Centre, Liverpool, UK; d Faculty of Medicine, University of Liverpool, Liverpool, UK

**Keywords:** Course, Interview, Virtual

Dear Editor,

Entry into neurosurgical training in the UK is amongst the most competitive applications across all medical and surgical specialties. Entry points at ST1 and ST2 levels saw competition ratios of 16.13 and 59.00 in 2021 [1], respectively. In comparison, ratios for Core Surgical Training and Internal Medicine Training were 4.16 and 2.29, respectively. In addition, given the ongoing difficulties with workforce planning in Neurosurgery, the number of available posts have reduced dramatically over the last few years from 34 in 2018 to 15 in 2022. Despite increasing levels of competition, there is a paucity of formal interview guidance for prospective applicants at the undergraduate and early postgraduate stages. The result is a mandatory reliance on direct contact with the neurosurgical community, which is not always possible due to geographical constraints.

To address this, and the additional complexity introduced by the advent of virtual interviews, we designed the first nationally run virtual interview course for neurosurgery applicants. The course consisted of morning and afternoon segments. The morning involved three didactic lecture sessions on the clinical, management, and portfolio stations. The afternoon consisted of three 1:1 interviews between applicants and faculty. Each station lasted 15 min with a 5-min break to mimic national selection standards. Each candidate was provided with a pre- and post-course survey questionnaire to identify further details regarding demographics, training grade, confidence, and perceived knowledge of common neurosurgical conditions.

A total of 25 candidates attended the course, of which 44% were female; an encouraging figure when compared to the proportion of female consultants in the UK (3%) [2]. Gender parity needs to be encouraged by optimising course advertisement, course timings, and course content [3]. Most candidates were Foundation Year 2 level or senior (80%). 60% of participants did not have a neurosurgical centre attached to their medical school or base hospital. Overall, post-course feedback reported substantial benefit, with improvement in understanding of interview structure, confidence in approaching clinical and management scenarios, and appreciation of pertinent aspects of leadership and management within the NHS. Importantly, perceived confidence with interviews via virtual methods was very high.

With national selection entry further constrained in 2022, there is increasing pressure to ensure a fair and transparent process for prospective applicants. Our course encouraged relatively high female participation and geographically democratised access to national selection preparation material. Furthermore, the curriculum encouraged wider knowledge of the NHS, and improved candidates understanding of neurosurgery as a speciality. Most importantly, the mock interviews displayed the candidate's strengths and weaknesses, thereby encouraging reflective practice. With the guided practice our course offered, we aim to increase the preparedness of candidates for the UK national selection process. Furthermore, we hope that this enhances the interview experience for both interviewers and interviewees alike. We aim to run annual courses, either face-to-face or virtual, with the help of the neurosurgical trainees across the UK to continue providing a point of contact and support for applicants.

## Funding

No funding was sought for this study.

## Declaration of competing interest

The authors report there are no competing interests to declare.
